# β1 Integrin-Focal Adhesion Kinase (FAK) Signaling Modulates Retinal Ganglion Cell (RGC) Survival

**DOI:** 10.1371/journal.pone.0048332

**Published:** 2012-10-31

**Authors:** Andrea Rachelle C. Santos, Raul G. Corredor, Betty Albo Obeso, Ephraim F. Trakhtenberg, Ying Wang, Jamie Ponmattam, Galina Dvoriantchikova, Dmitry Ivanov, Valery I. Shestopalov, Jeffrey L. Goldberg, Mary Elizabeth Fini, Michaela Livia Bajenaru

**Affiliations:** 1 Bascom Palmer Eye Institute, University of Miami Miller School of Medicine, Miami, Florida, United States of America; 2 Neuroscience Program, Interdisciplinary Stem Cell Institute, University of Miami Miller School of Medicine, Miami, Florida, United States of America; 3 Institute for Genetic Medicine, Keck School of Medicine, University of Southern California, Los Angeles, California, United States of America; Dalhousie University, Canada

## Abstract

Extracellular matrix (ECM) integrity in the central nervous system (CNS) is essential for neuronal homeostasis. Signals from the ECM are transmitted to neurons through integrins, a family of cell surface receptors that mediate cell attachment to ECM. We have previously established a causal link between the activation of the matrix metalloproteinase-9 (MMP-9), degradation of laminin in the ECM of retinal ganglion cells (RGCs), and RGC death in a mouse model of retinal ischemia-reperfusion injury (RIRI). Here we investigated the role of laminin-integrin signaling in RGC survival in vitro, and after ischemia in vivo. In purified primary rat RGCs, stimulation of the β1 integrin receptor with laminin, or agonist antibodies enhanced RGC survival in correlation with activation of β1 integrin’s major downstream regulator, focal adhesion kinase (FAK). Furthermore, β1 integrin binding and FAK activation were required for RGCs’ survival response to laminin. Finally, in vivo after RIRI, we observed an up-regulation of MMP-9, proteolytic degradation of laminin, decreased RGC expression of β1 integrin, FAK and Akt dephosphorylation, and reduced expression of the pro-survival molecule bcl-xL in the period preceding RGC apoptosis. RGC death was prevented, in the context of laminin degradation, by maintaining β1 integrin activation with agonist antibodies. Thus, disruption of homeostatic RGC-laminin interaction and signaling leads to cell death after retinal ischemia, and maintaining integrin activation may be a therapeutic approach to neuroprotection.

## Introduction

The ECM protein laminin is present in many areas of the CNS, particularly during development. However, laminin continues to be an important, abundant component of the ECM in adult CNS. Laminins are critical to neurite outgrowth and to the structure of the neuronal synapse [1]. They are also a major component of ECM and basement membranes in the retina [2], being involved in RGC migration and subsequent development [3]. Laminin chains α2 [4], and β1 [5] have been directly associated with RGCs in the retina. Together with growth factors, laminin promotes survival and axon outgrowth in purified RGCs in culture [6].

We and others have shown that laminin degradation after a variety of insults including retinal ischemia, and glaucoma is associated with up-regulation of a specific matrix metalloproteinase, MMP-9, and decreased RGC survival in those animal models [7]–[10]. MMP-9 knockout mice, or mice treated with MMP inhibitors demonstrate reduced RGC death after injury, suggesting a key role for the up-regulation of MMP-9 in promoting RGC death [7]–[9].

Signals from ECM are transmitted to neurons through integrins, a family of heterodimeric receptors that mediate cell to cell, and cell to ECM interactions [11], [12]. The combination of α and β subunits determines integrin ligand specificity and intracellular signaling activity [13]. Integrins have a variety of cellular functions including cell shape changes, motility, survival, and proliferation. They regulate cell survival either directly by binding to ECM proteins, or indirectly by interacting with growth factors’ signaling pathways [14]. Beyond maintaining homeostatic cellular function, integrins are implicated in cellular resistance to apoptotic stimuli [15].

In the present study, we investigated the role of laminin-integrin signaling in RGC survival, and protection against ischemic injury. We tested the hypothesis that up-regulation of MMP-9 following RIRI, and subsequent laminin degradation results in loss of signaling through the integrin survival pathway, leading to RGC death. RGCs are excellent models to study mechanisms of neuronal survival, and neuroprotection, because they are CNS neurons accessible outside the cranial cavity for quantitation, and manipulation. We performed integrin survival signaling studies in a rat RIRI model, clinically relevant to neuronal injury. RIRI occurs in a variety of retinal conditions including central retinal artery (CRAO), and central vein occlusion (CRVO), proliferative diabetic retinopathy, glaucoma, and can cause sudden irreversible vision loss. We also tested the ability of a β1 integrin activating antibody [16] to mimic the survival effect of laminin in RGC in culture, and successfully used this stimulatory strategy, to rescue homeostatic matrix-integrin signaling, and enhance RGC survival in vivo in the rat RIRI model. Maintaining β1 integrin activated at the cell surface might represent a valuable therapeutic approach for ischemic-reperfusion injury.

## Materials and Methods

### Ethics Statement

Animal use strictly followed the guidelines of the ARVO statement for the use of Animals in Ophthalmic and Vision Research as well as the Policies on the Use of Animals in Neuroscience Research. The protocol was approved by the Institutional Animal Care and Use Committee (Protocol Number: 11–263).

### Animals

Adult female Sprague-Dawley rats (250–300 g) were obtained from Harlan Laboratories, Inc. (Allen Park, MI) and were housed under a 12 hour light-dark cycle with access to food and water ad libitum. The animals were anesthetized with an intraperitoneal injection of a cocktail of Ketamine 60 mg/kg and Xylazine 7.5 mg/kg prior to the start of the experiment.

### Rat Retinal Ischemia-reperfusion Model Injury (RIRI)

In this model, RIRI was achieved by unilateral intraocular pressure elevation through cannulation of the anterior chamber with a 30-gauge needle connected to a saline (0.9% NaCl) reservoir placed above the rat eye, with an applied pressure of 110–120 mm Hg. The intraocular pressure was measured with a tonometer (Biophysics Ophthalmic Unit, Bascom Palmer Eye Institute, University of Miami, FL). The elevated intraocular pressure was maintained for an hour. After removal of the needle and saline reservoir, the IOP will decrease to normal values in 5 min and reperfusion occurs [17]. The contralateral eye served as a control eye and was cannulated similarly but the reservoir was maintained at the level of the eye and there was no flow of saline in the anterior chamber. Complete retinal ischemia was verified in each animal under microscopic examination by the whitening of the anterior segment of the eye and blanching of the retinal arteries. Core body temperature was maintained throughout the procedure at 37°C with a heating pad. Groups of animals were sacrificed at various time points after reperfusion, and their eyes were enucleated. The retinas were separated and were processed for RGC counting, as well as immunocytochemistry, and biochemical studies.

### Retrograde Labeling of RGC and Quantitation of RGC Death

Specific labeling of the RGC was acquired by bilateral retrograde labeling with neuronal tracers. One week before ischemia, Fluoro-Gold (FG) (Fluorochrome, LLC, Denver, CO) (2% in 0.9% NaCl containing 10% dimethyl sulfoxide) or 4DI-10 ASP (DiA) (3% in ethanol) were microinjected bilaterally with a total volume of 6 µl with a Hamilton syringe (Stoelting, Wood Dale, IL) into the superior colliculi of the rat using a stereotactic equipment (Stoelting). RGC loss was quantified after unilateral injury by comparing the experimental eye to the contralateral control eye of the same animal. Rats were euthanized at 1 and 5 days by intra-cardial perfusion with 4% paraformaldehyde and both the left (ischemia) and right (control) retinas were dissected, fixed for an additional 30 minutes in 4% paraformaldehyde, and flat mounted vitreal side up on a glass slide for examination of the ganglion cell layer to assess RGC death. The FG labeled RGC were examined by fluorescence microscopy in a UV wide band, with a Carl Zeiss Axiovert 200 M inverted microscope equipped with an AxioCam MR monochrome digital camera (Carl Zeiss, Gottingen, Germany). Pictures of a total of 32 areas, 8 in the central, 8 in the middle, and 16 in the peripheral retina were taken at 400X magnification, and FG labeled RGCs were counted manually in these pictures by two investigators in a masked fashion. Initially, the scores were averaged and expressed per central, middle, and peripheral retina. Because differences in RGC death between central, middle, and peripheral areas, at each time point, were not statistically significant, for the HUTS-21 activating antibody treatment experiment, the number of FG labeled RGCs counted in 32 areas were averaged, and expressed per whole retina. Changes in RGC density were expressed as percentage RGC survival in the ischemic eye, normalized to the control contra-lateral eye from the same animal [18]–[20].

### TUNEL Staining

RGC apoptosis was monitored in the rat retina in frozen sections by using a terminal deoxynucleotidyl transferase (TdT)-dUTP nick-end labeling (TUNEL) in situ cell death detection kit (Roche Applied Science, Indianapolis, IN) according to manufacturer’s instructions.

### β1 Integrin Activating Antibody (HUTS-21) *in vivo* Treatment

RIRI was induced in 20 Sprague-Dawley rats as described above. The animals were divided into 4 groups: non-treated (n = 4), HUTS-21 antibody treated (n = 6), isotype control antibody (n = 6), and intravitreal injection PBS control (n = 4). 5 µl β1 integrin activating antibody (HUTS-21) or isotype control antibody (0.5 mg/ml; BD Pharmingen, San Diego, CA), or PBS were administered intravitreally to the rat eye twice: 30 minutes, and 2 days post-RIRI. Animals were euthanized 5 days after RIRI and FG labeled RGC were counted in flat mounted retinas.

### In Situ Zymography

Following enucleation, fresh, unfixed rat eyes (n = 10) were embedded in Optimal Cutting Temperature (OCT) (Sakura Finetek, Torrance, CA), flash frozen in liquid nitrogen, and serially sectioned (8 µm). Sections were incubated with (FITC)-labeled DQ-gelatin (fluorescein isothiocyanate-labeled dye-quenched-gelatin; 40 mg/ml; Molecular Probes, Eugene, OR) in 1X reaction buffer (Molecular Probes) overnight at 37°C in a humidity chamber. Sections were rinsed three times in PBS and mounted in Vectashield with DAPI (4,6-diaminidino-2-phenylindole) (Vector Laboratories, Burlingame, CA) for fluorescence microscopy. DQ-gelatin is gelatin heavily labeled with FITC molecules, so its fluorescence is quenched. When DQ-gelatin is cleaved by gelatinolytic activity, fluorescent peptides are released and become visible against a weakly fluorescent background representing net proteolytic activity [21].

### Tissue Processing and Immunohistochemistry

Rats were perfused intra-cardially with 4% paraformaldehyde in 0.1 M phosphate buffer, pH 7.4, and the rat eyes were enucleated at 1 hour, 6 hours, 24 hours, and 5 days post RIRI and fixed immediately in 4% paraformaldehyde overnight. The anterior part of the eye and lens were removed, and the remaining eye cup was equilibrated in graded sucrose solutions (10–30% in PB) for several hours at 4°C. They were then embedded in OCT and frozen in liquid nitrogen. Radial cryosections (8 µm) were collected and mounted onto glass slides coated with poly-L-lysine and processed. The retinal sections were incubated in PBS Blocking Buffer (1% BSA, 0.2% milk, 0.3% Triton X-100) for 30 minutes at room temperature. Each primary antibody was incubated in the same blocking buffer overnight at 4°C. Sections were then incubated with the appropriate secondary antibody for an hour at room temperature, washed with PBS, and mounted in Vectashield mounting medium for fluorescence with DAPI. The antibodies used are as follows: anti-β1 integrin (Millipore, Billerica, MA) 1/100, anti-FAK (BD Biosciences, San Jose, CA) 1/100, anti-FAK [pY-397] (Invitrogen, Camarillo, CA) 1/100.

For immunohistochemistry of purified RGC in culture, cells were fixed with 4% paraformaldehyde. Cultures were blocked and permeabilized with 10% normal goat serum (NGS) and 0.2% Triton X-100 in PBS. They were incubated overnight at 4°C with primary antibodies in 10% NGS in PBS and secondary antibodies with the same buffer for 4 hours at room temperature. The following primary antibodies were used: β1 integrin 1/100, P-FAK 1/100 and FAK 1/50, and neuronal class III β-tubulin (TUJ1; Covance, Emeryville, CA) 1/300. Alexa Fluor-488, -568, -647 highly cross-adsorbed antibodies (Invitrogen, Carlsbad, CA) 1/500 were used as secondary antibodies. Immunohistochemistry was visualized by fluorescent microscopy (Zeiss Axiovert), and laser confocal microscopy (Leica TCP SP5; Wetzlar, Germany).

### Primary Culture of RGCs

For in vitro studies, one litter of postnatal day (P5) Sprague-Dawley rats was used. The reagents were purchased from Sigma-Aldrich, St. Louis, MO, unless otherwise specified. The retinas were purified through sequential immunopanning to 99.5% purity and cultured on poly-D-lysine (PDL; 70 kDa, 10 µg/ml), or PDL and laminin (1 µg/ml) (Invitrogen, Carlsbad, CA) coated 8 well chamber slides (Lab-Tek II chamber slide with cover, Naperville, IL) in neurobasal medium (NB) (Invitrogen, Carlsbad, CA), containing L-glutamine (1 mM; Gibco, Invitrogen, Carlsbad, CA), triido-thyroxine (T3; 40 ng/mL) insulin (5 µg/mL), sodium pyruvate (1 mM; Invitrogen, Carlsbad, CA), N-acetyl cysteine (NAC; 5 µg/mL), Pen/Strep and B27 (Gibco, Invitrogen, Carlsbad, CA), Sato supplement, BDNF (50 µg/mL), CNTF (10 µg/mL) and Forskolin (5 µM) as previously described [22]–[24].

### Cell Treatments

β1 integrin activating, HUTS-21, or isotype control rat antibodies, (0.5 mg/ml; BD Pharmingen, San Diego, CA), were added to cultured RGCs 12 hours after plating to a final concentration of 1 µg/ml and incubated for 24, and 48 hours.

A selective inhibitor of the Src family of protein tyrosine kinases, PP2 [4-Amino-5-(4-chlorophenyl)-7-(*t*-butyl) pyrazolo-[3, 4-d] pyrimidine] (Calbiochem, San Diego, CA) (25 µM) was added to RGCs immediately after plating and incubated for 48, and 72 hours.

### siRNA Knockdown

Purified RGCs from P3–P4 Sprague-Dawley pups were transfected by electroporation with FAK siRNA 5′-AGAAAUAGCUGAUCAAGUAdTdt-3′ or non-targeting control siRNA (Dharmacon, Inc., Lafayette, CO) using the Amaxa Nucleofactor II (Lonza, Cologne, Germany) device. 300,000 cells, resuspended in 27 µl of transfection medium and 1 µl of FAK siRNA, or control siRNA were placed in a cuvette (Sigma-Aldrich, St. Louis, MO) and electroporated using the Amaxa program SCN#1. After electroporation, cells were resuspended in growth medium, centrifuged for 16 minutes at 1800 rpm, and plated in triplicates at 10^4^ cells/1.0 cm^2^ in 48-well plates for survival and neurite growth assays, at 2×10^5^ cells/3.2 cm^2^ well in 12-well plates for real-time PCR (RT-PCR) and 5×10^3^ cells in 8 well chamber slides for immunohistochemistry, on PDL/laminin coated plates and incubated for 24, or 72 hours.

### RT-PCR

Total RNA was extracted from RGCs using Nanoprep (Stratagene, Carlsbad, CA) and reverse transcribed with Superscript III polymerase (Invitrogen, Grand Island, NY) to synthesize cDNA. RT-PCR was performed with the Rotor-Gene 6000 Cycler (Qiagen, Valencia, CA) using the SYBR Green PCR MasterMix (Qiagen, Valencia, CA). Knockdown of FAK in RGC was tested with primers specific for FAK, forward: 5′-AAAATGTGACGGGCCTAGTG-3′, and reverse 5′-TACTCCTGCTGAAGGCTGGT-3′. Quantification of FAK expression was calculated by comparison with a standard curve following normalization to 18S; forward: 5′-GAACTGAGGCCATGATTAAGAG-3′ and reverse: 5′-CATTCTTGGCAAATGCTTTC-3′.

### RGC Survival and Neurite Counting

RGC survival was assessed by live cell imaging with an Axiovert Zeiss 200 M microscope, equipped with an environmental chamber for temperature, and CO_2_ control, using a motorized stage with a coordinate system to localize the same areas in a given well at 0, 24, and 48 hours. Cells were cultured on PDL, or PDL/laminin coated 8 well chamber slides (Lab-Tek II chamber slide with cover, Naperville, IL) at a density of 10^4^ cells/well as described above. 21 bright-field random pictures were acquired for each well. They were analyzed with Axio Vision 4.7 software to manually determine the percentage of RGCs that were still alive at the end of the experiment (t = 24, or 48 hours) when compared to the beginning of the experiment (t = 0). RGC survival was determined by manually counting 100–500 live cells in the acquired micrographs.

For neurite length and survival assays after FAK siRNA knock-down, RGCs were fixed with 4% PFA for 30 minutes, permeabilized in 0.2% Triton X-100 along with 10% NGS in antibody buffer for 30 minutes, and immunostained with neuronal class III β-tubulin 1/300 (Covance, Emeryville, CA), DAPI 1/300 and Alexa Fluor 488 1/500 (Invitrogen) secondary antibodies. Condensed, bright DAPI positive cells with fragmented or rounded cell bodies with no, or very short neurites were indicative of dead cells. RGC survival was analyzed by counting percent of TUJ1/DAPI (neuronal class III β-tubulin) positive live cells out of total DAPI count in 3 wells/experimental condition in total of 3 experiments.

Average total neurite length per neuron was evaluated by manually tracing and measuring neurites which were at least two cell bodies in length using Axio Vision 4.7 software, or using High Content Analysis system and Kineticscan software (Cellomics, Pittsburgh, PA).

### Western Blotting

Fresh retinas were homogenized in NP40 lysis buffer (Invitrogen, Camarillo, CA) containing protease inhibitors (Complete Mini; Roche, Indianapolis, IN) and phosphatase inhibitors cocktail (Thermo Scientific, Rockford, IL). The protein concentration was determined by Bradford protein assay (BioTek, Winooski, VT). Retinal extracts (60 µg) were separated on 10% SDS polyacrylamide gels (NuSep, Bogart, GA) and transferred to nitrocellulose filters. To block nonspecific binding, filters were placed in 10 mM Tris, pH 8.0, 150 mM NaCl, 0.2% Tween 20 (TBS-T), and 5% dry skim milk for 1 hour at room temperature. Blots were then incubated overnight at 4°C with the following primary antibodies: anti-integrin β1, anti-FAK, anti-FAK [pY-397], Akt, P-Akt (Ser473), Bcl-xL, Bcl-2 (Cell Signaling, Danvers, MA) and laminin (pan-laminin) (Sigma, St. Louis, MO). The polyclonal anti-laminin antibody was raised using as an immunogen laminin purified from the basement membrane of the Engelbreth Holm-Swarm (EHS) sarcoma. The most abundant laminin in EHS is laminin-1. Membranes were then washed in TBST and incubated in horseradish peroxidase (HRP)-conjugated anti-mouse or anti-rabbit IgG (0.5 ug/ml; Amersham Biosciences, GE Healthcare, Piscataway, NJ) for one hour at room temperature. Blots were developed and analyzed using the Fujifilm LAS-4000 software (GE Healthcare, Piscataway, NJ). Quantitation was done through Image J and Multigauge Fujifilm LAS-4000 software. All samples were normalized to β-actin (Cell Signaling, Danvers, MA).

### Statistical Analysis

In all quantification experiments results are expressed as means ± S.D. Statistical analysis was performed with Student’s *t*-test. All statistical calculations were performed using SPSS 16.0 software (IBM Corporation, Armonk, NY). A p<0.05 was considered statistically significant (* = p<0.05; ** = p<0.01).

## Results

### Retinal Ischemia Leads to RGC Apoptosis

**Figure 1:**
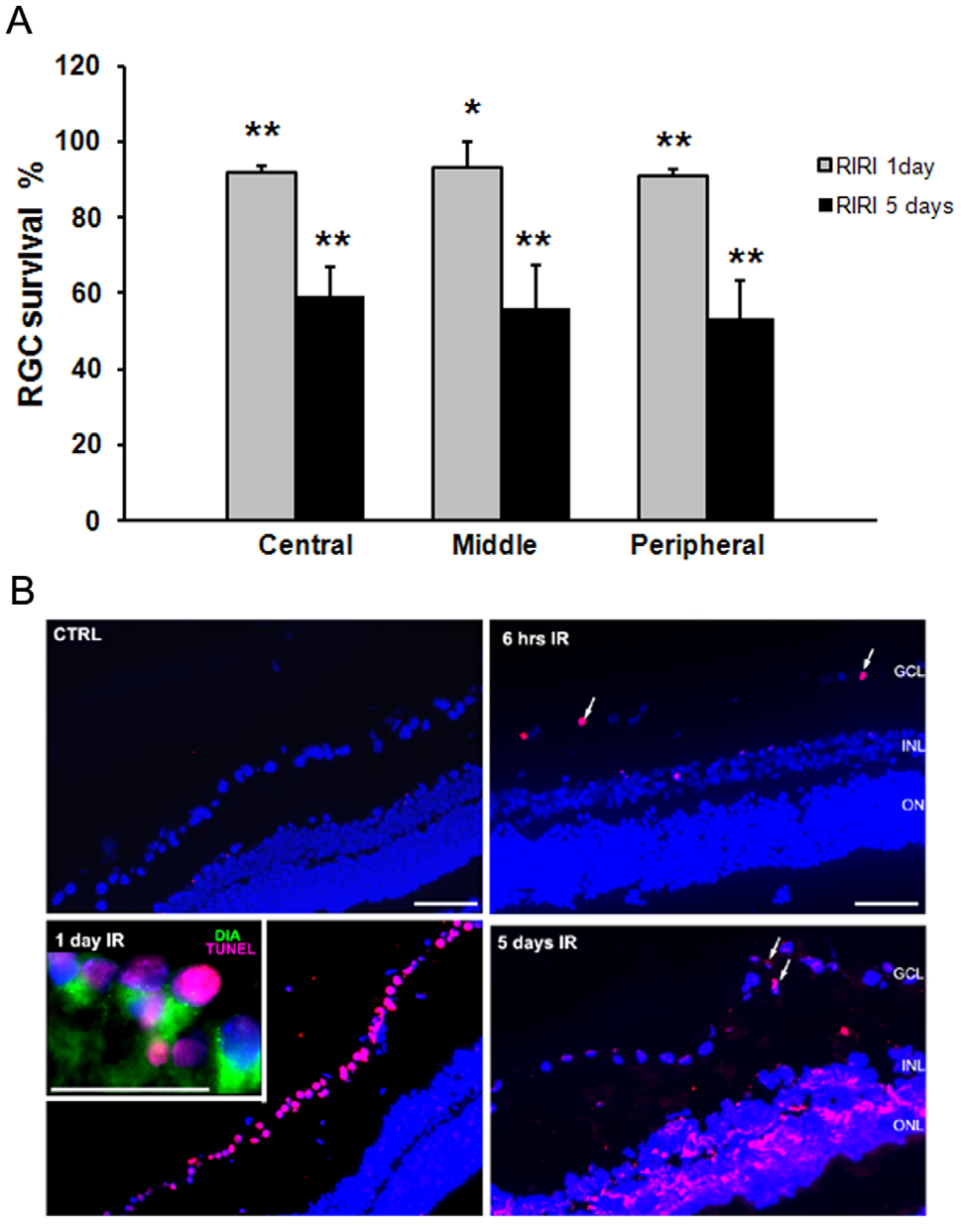
Retinal ischemia leads to RGC apoptotic cell death. **A.** Quantitation of FG labeled surviving RGCs at 1, and 5 days post-RIRI. Changes in RGC density were expressed as percentage RGC survival in the ischemic eye, normalized to the control contra-lateral eye from the same animal (n = 8 were analyzed for each time point). Student’s *t*-test. **p<0.01, *p<0.05. **C.** TUNEL staining in control (CTRL), and ischemic (IR) retina at 6 hours, 1, and 5 day post-injury. Note that larger magnification inset shows that most of the TUNEL positive cells (purple) in the GCL 1 day post-RIRI are RGC as demonstrated by co-localization with DiA retrograde label (green). Cellular nuclei are stained with DAPI (blue). Scale bars: 100 µm. GCL = ganglion cell layer, INL = inner nuclear layer, and ONL = outer nuclear layer. (n = 4 animals for each time point).

To quantify RGC loss in the rat retina after RIRI, we retrogradely labeled RGCs with FG, subjected the retina to RIRI, and then counted FG-labeled RGCs in the central, middle, and peripheral retina. RGC loss was uniform throughout the retina with no significant differences between the central, middle, and peripheral retina. We found 40.68% (**p<0.01), 44.07% (**p<0.01), and 46.43% (**p<0.01) RGC loss in the central, middle, respectively peripheral ischemic retina compared with control at 5 days post-RIRI; RGC loss 1 day post-RIRI was minimal, but statistically significant in the central, 7.97%, (**p<0.01), middle, 6.98% (*p<0.05), and peripheral 8.96% (**p<0.01) ischemic retina (Fig 1A).

Apoptosis is involved in neuronal cell death in animal models of transient retinal ischemia and has been detected in the ganglion cell layer (GCL), inner nuclear cell layer (INL), and the outer nuclear layer (ONL) of the retina [25]–[28]. To assess RGC apoptosis, we performed TUNEL (terminal deoxynucleotidyl transferase-mediated dUTP nick-end labeling) in retinal sections from ischemic and control eyes at 1, 6, 24 hours, and 5 days post-RIRI. TUNEL positive cells were noticeable in the GCL at 6 hours, and reached a peak at 24 hours post-RIRI. This was not evident in the control eye (Fig 1C). Most of the TUNEL positive cells in the GCL 24 hours post-RIRI were RGCs as demonstrated by co-localization with a DiA retrograde label (Fig 1C, inset). Thus, RGC apoptotic death after RIRI peaked at 24 hours, and loss of RGCs was apparent at 5 days, suggesting a rapid time course of cell death after ischemic injury.

### Laminin Degradation is Associated with Increased MMP-9 Expression and Activity and RGC Apoptosis after RIRI

**Figure 2:**
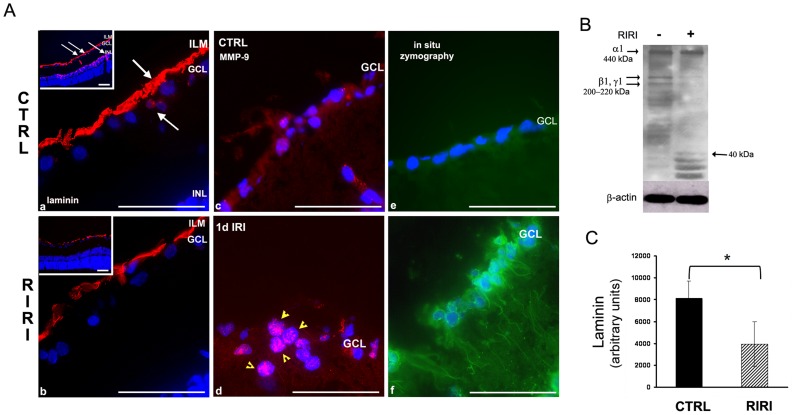
Laminin degradation is associated with increased MMP-9 expression and activity in the retina 1 day after RIRI. **A.** Immunofluorescence with a pan-laminin antibody in retinal sections from control (CTRL), and ischemic (RIRI) eyes shows laminin expression (red) in the inner limiting membrane (ILM), ganglion cell layer (GCL), and inner nuclear layer (INL) (**a, b**, and lower magnification insets). White arrows in **a,** and corresponding lower magnification inset point toward sites of laminin degradation in the ECM of RGC, in the ILM, around the RGC cell body, and INL. Note thinning of laminin in the ILM, and loss of laminin expression in the RGC and INL after RIRI. (**b,** and lower magnification inset). Immunofluorescence with anti-MMP-9 antibodies shows increased expression of MMP-9 (red, yellow arrows; **c, d**), and in situ zymography demonstrates increased gelatinolytic activity (green) (**e, f**) in the ganglion cell layer (GCL) in ischemic eyes (n = 12). **B**. Western blotting of retinal extracts of control (−), and ischemic (+) rat eyes 1 day post-RIRI (n = 3). **C**. Densitometric analysis of bands corresponding to laminin β1 and γ1 chains in control and ischemic eyes normalized to β-actin. Error bars, SD. Student’s *t* test. *p<0.05. Scale bars: 100 µm.

We next asked whether changes in laminin and MMP-9 are associated with RIRI. To investigate laminin degradation in the rat RIRI model, retinal sections were analyzed by immunofluorescence with a polyclonal pan-laminin antibody at 1, 6, 24 hours and 5 days post-RIRI. Laminin degradation was detected 24 hours post-RIRI in the ECM surrounding RGCs, in the inner limiting membrane (ILM), GCL, and INL, where RGCs establish connections with other neurons (Fig 2A a, b and insets). Further degradation of laminin was observed 5 days post-RIRI, while at earlier time points 1, and 6 hours no laminin degradation was observed (data not shown). The expression of MMP-9 was analyzed by immunofluorescence with anti-MMP-9 antibodies, and the gelatinolytic activity with the fluorogenic DQ gelatin substrate in frozen retinal sections from ischemic, and control eyes at 1, 6, 24 hours, and 5 days post-RIRI. We found increased expression of MMP-9 (Fig 2A c, d), and gelatinolytic activity (Fig 2A e, f) in the GCL, clearly co-localized with RGCs, 1 day post-RIRI. Cells in the inner plexiform layer (IPL), expressing MMP-9 (Fig 2A c) are either Muller glia [29], or vascular endothelial cells [30], shown to express MMP-9 in the normal retina. RIRI leads to a significant increase in gelatinolytic activity as early as 6 hours post-injury preceding the proteolytic degradation of laminin (data not shown). This is in correlation with our previous western blotting and gel zymography data showing that MMP-9 expression and activity increases over a time of 6–24 hours [7].

Changes in laminin expression were quantitated by western blotting with pan-laminin antibodies in retinal extracts from ischemic and control eyes at 1, 6, 24 hours post-RIRI. Laminin 1 was detected by the very prominent α1 chain at 440 kDa, and β1, γ1 chains at 200–220 kDa [31], [32] (Fig 2B). However, this antibody will detect all other laminins that contain β1, γ1 chains, such as the ILM laminin 10 (α5β1γ1), laminin 2 (α2β1γ1) produced by RGCs, and laminin 4 (α4β1γ1), a component of the ECM of the INL. Laminin degradation was detected after RIRI by decreased intensity primarily of the 200–220 kDa bands corresponding to laminin chains β1 and γ1, while the α1 chain remained intact. In addition, proteolytic laminin fragments of 40 kDa and below appeared in the lane corresponding to the ischemic retina (Fig 2B). Quantitation of the β1 and γ1 bands showed a 2-fold decrease in laminin expression in the ischemic eyes at 1 day post-RIRI (Fig 2C). In contrast, at 1, and 6 hours post-RIRI, there was no significant decrease in the β1 and the γ1 bands, indicating that proteolytic degradation of laminin in the ECM of RGCs started between 6–24 hours after retinal ischemic injury (data not shown).

Our current, and previous results [7] show that the decrease in laminin expression is spatially and temporally correlated with an increase in MMP-9 expression, and activity 24 hours after RIRI. The increase in MMP-9 activity in the ECM of RGC starts as early as 6 hours post-injury, and is followed by proteolytic degradation of laminin between 6–24 hours. MMP-9 up-regulation, and subsequent laminin degradation were prominent at 24 hours when we detected a peak of RGCs apoptosis, and occurred before RGC loss in the RIRI model.

### Integrin Signaling is Down-regulated in RGCs in the Rat Retina after Ischemic Injury

**Figure 3:**
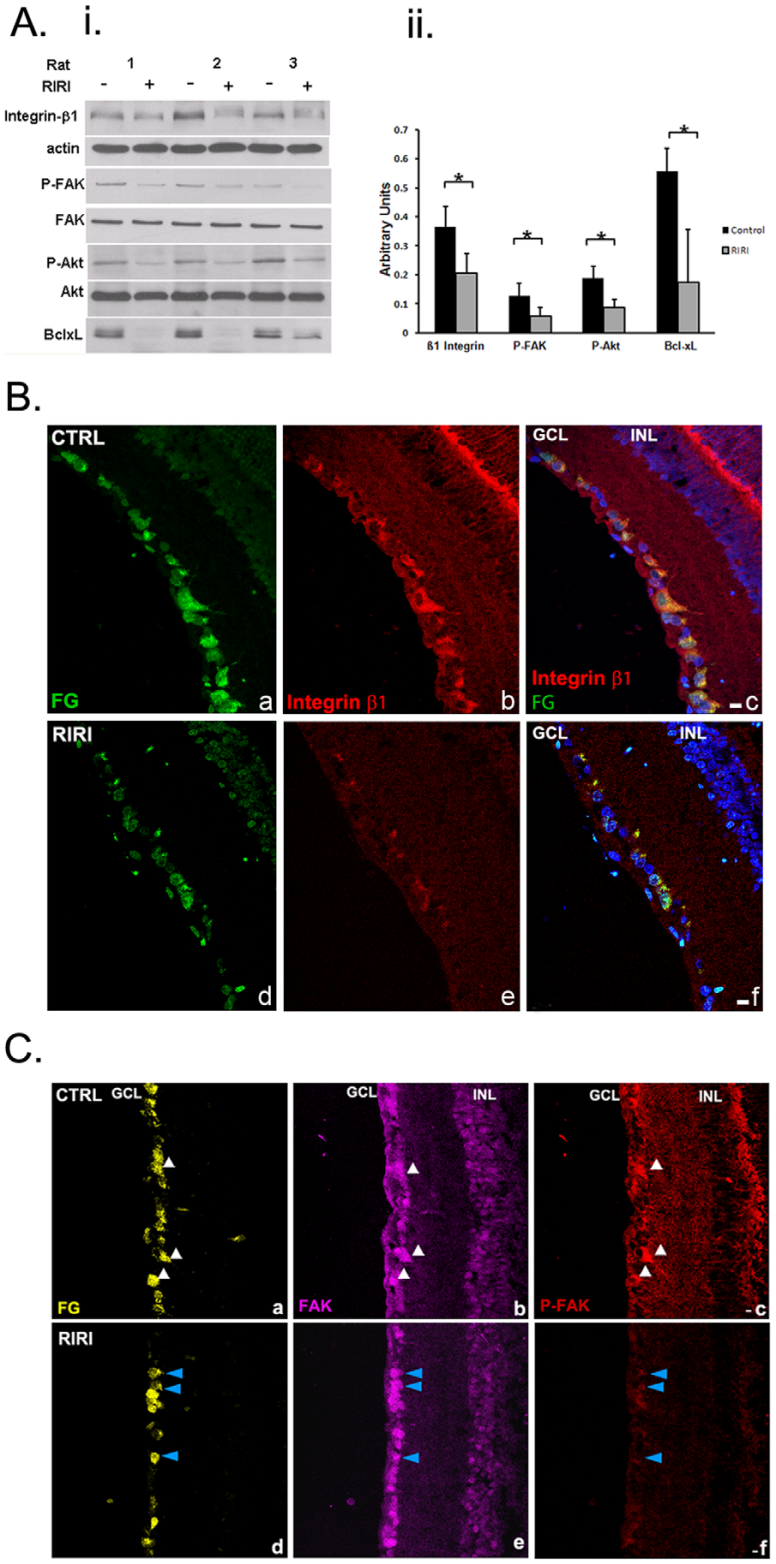
Integrin survival signaling is down-regulated in RGC in the retina 1 day after ischemic injury. A. (**i** ) Western blot of retinal extracts of ischemic, RIRI (+), and control (−) rat eyes 1 day post-injury (n = 3). A representative example of at least 5 independent experiments is presented. (**ii**) Densitometric analysis of western blotting with all samples normalized to β-actin. Error bars, SD. Student’s *t* test. *p<0.05. **B.** β1 integrin expression is decreased in RGC 1 day post-RIRI. Immunohistochemistry with β1 integrin antibodies (red) in frozen retinal sections from control (**a, b, c**), and ischemic retinas (**d, e, f**), in which RGCs were retrogradely labeled with FG (green; **a, d**) shows reduced β1 integrin expression in ischemic eyes in comparison with control eyes (red; **b, e**) specifically in RGC (yellow; merged; **c, f**). Nuclei are stained with DAPI (blue; **c, f**). (n = 12). GCL = ganglion cell layer, INL = inner nuclear layer. Scale bars: 10 µm. **C.** FAK activation is reduced in RGC 1 day after ischemia. Immunohistochemistry with FAK (purple), and P-FAK [pY^397^] (red) antibodies in frozen retinal sections of control (**a, b, c)**, and ischemic retinas (**d, e, f**) at 1 day post-RIRI, in which RGCs were retrogradely labeled with FG (yellow; **a, d**) shows significantly decreased expression of the activated, phospho [pY^397^] FAK in ischemic eyes in comparison with control eyes (red; **c, f**), while FAK expression remains unchanged (purple; **b, e**). White arrows identify FG labeled RGCs in the control retina (CTRL) expressing high levels of both FAK, and P-FAK [pY^397^] (**a, b, c**). Blue arrows point toward RGCs in the ischemic retina (RIRI) that have markedly decreased expression of P-FAK [pY^397^], while FAK expression is the same (**d, e, f**). (n = 12). GCL = ganglion cell layer, INL = inner nuclear layer. Scale bars: 10 µm.

Since proteolytic degradation of laminin occurs after ischemic reperfusion injury, and is linked to RGC death, and integrins are the main receptors for ECM, we next tested the hypothesis that integrin survival signaling is disrupted in RGCs. We performed Western blotting analysis of retinal extracts from ischemic and control eyes at 1 day post-injury (Fig 3A). Densitometry revealed a statistically significant 1.8-fold decrease in β1 integrin expression in ischemic eyes in comparison with control eyes (Fig 3Ai, and ii). β1 integrin signaling requires in many cells types activation of the non-receptor tyrosine kinase, focal adhesion kinase (FAK) [33]. Upon integrin binding to ECM, FAK becomes activated by undergoing an initial autophosphorylation at the Tyr 397 residue [34]. Our analysis showed that RIRI caused a 2-fold decrease in phosphorylation of FAK at Tyr 397, while total FAK expression remained unchanged (Fig 3Ai, ii). FAK initial activation by auto-phosphorylation at Tyr 397 is followed by phosphorylation at multiple amino acids [35] involved in recruitment and activation, mainly by phosphorylation of several classes of signaling molecules, including the survival molecule Akt [36], [37]. Western blotting of retinal extracts 1 day after ischemic injury showed more than 2-fold dephosphorylation of Akt at Ser 473, while total Akt expression remained unchanged (Fig 3Ai, ii). In addition, there was a 4-fold decrease in the anti-apoptotic protein bcl-xL (Fig 3Ai, ii). There were no changes in β1 integrin expression, and [pY397] FAK at 6 hours post-ischemia (not shown).

To show that integrin signaling is disrupted specifically in RGCs 1 day after RIRI, we performed immunohistochemistry in frozen retinal sections from rats in which the RGCs were retrogradely labeled with FG. Confocal microscopy analysis of immunohistochemistry with anti-integrin β1 antibodies showed high expression of β1 integrin in the GCL (Fig 3B, b) and most of the expressing cells were FG-positive RGC (3B, c). Integrin β1 expression in RGCs was significantly reduced after ischemia (Fig 3B, b, e, and c, f).

FAK was widely expressed in the retina with particularly high expression levels in RGCs (Fig 3C a, b), which did not change after ischemia (Fig 3C, b, e). P-FAK [pY^397^] was expressed in RGCs in the cell body and neurites (Fig 3C, a, c). After ischemia P-FAK [pY^397^] expression was nearly undetectable, suggesting that FAK phosphorylation and subsequent activation is almost completely abolished (Fig 3C, c, f).

Our combined immunohistochemistry and Western blotting data strongly suggest that the integrin survival signaling pathway is disrupted in RGCs as a result of ischemia, and correlates with MMP-9 up-regulation, subsequent laminin degradation, and RGC apoptosis in the RIRI model.

### Laminin Adhesion Promotes Integrin Signaling and RGC Survival

**Figure 4:**
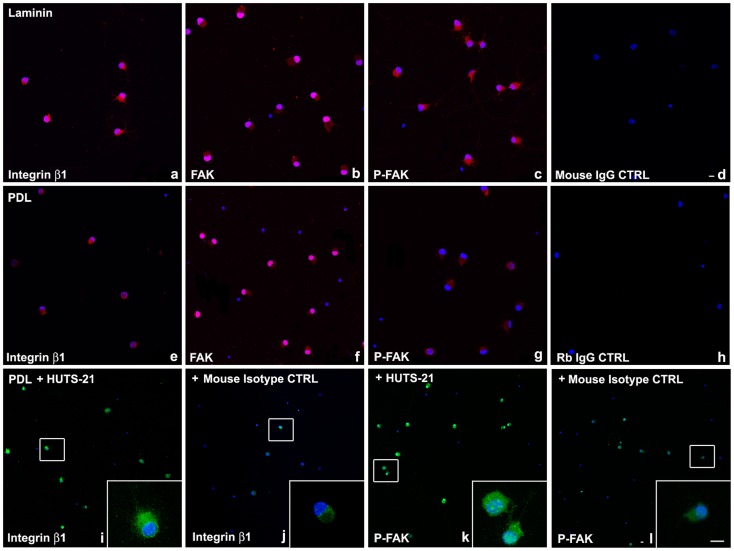
Laminin, or β1 integrin activating antibody, HUTS-21, promote integrin signaling in RGCs in vitro. P5 RGCs were cultured on laminin (**a–d**), or PDL (**e–l**) for 48 hours. HUTS-21 (**i, k**) or isotype control (**j, l**) antibodies (1 µg/ml) were added to RGCs cultured on PDL 12 hours after plating. Immunohistochemistry was performed with β1 integrin (**a, e, i, j**), FAK (**b, f**), [pY397]-FAK (**c, g, k, l**), or IgG control antibodies (**d, h**) and analyzed by confocal microscopy. 100–200 cells per condition were analyzed. A representative experiment is shown. A total of N = 5 experiments were performed. Scale bars: 10 µm.

To further explore the role of laminin-integrin signaling in RGC survival, we cultured purified RGCs on laminin and on poly-D-lysine (PDL) for 48 hours and performed immunohistochemistry with β1 integrin, FAK, and [pY397]-FAK antibodies. Confocal microscopy analysis showed that RGCs cultured on laminin have increased expression of β1 integrin receptors (Fig 4 a), compared to RGC on PDL (Fig 4 e). In addition the expression of phospho-[pY397]-FAK (active form) was dramatically increased in RGC on laminin (Fig 4 c), in comparison to RGC cultured on PDL (Fig 4 g), while total FAK expression was the same on both laminin, and PDL (Fig 4 b, f). RGC cultured on laminin (Fig 4 a, b, c), appeared morphologically larger, with more neurites, compared to RGC on PDL (Fig 4 e, f, g).

**Figure 5:**
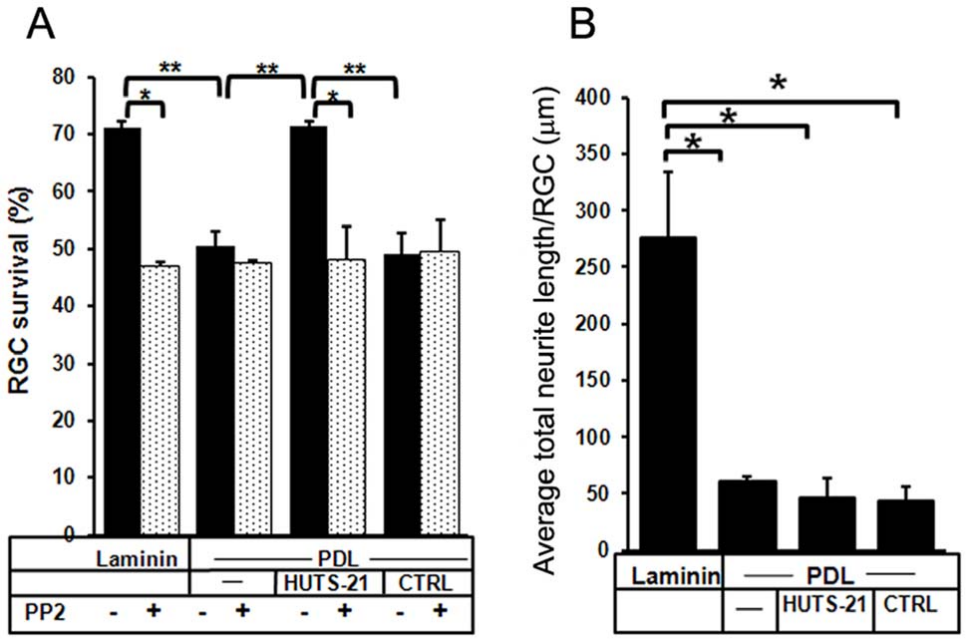
The agonist antibody HUTS-21 mimics laminin’s pro-survival effect in RGC. P5 purified RGCs were cultured on either laminin, or PDL for 48 hours. HUTS-21, or isotype control antibodies (1 µg/ml) were added to RGCs cultured on PDL 12 hours after plating. RGCs were pretreated with the Src/FAK inhibitor PP2 (25 µM). Live-cell imaging with an Axiovert Zeiss 200 M microscope was used to acquire micrographs of RGC in culture immediately after HUTS-21 addition, t = 0, and at the end of the experiment, 48 hours after plating, t = 48 hours. **A.** To evaluate RGC survival 100–500 live RGC/experimental condition were counted manually in the acquired micrographs to determine the percentage of live RGC at t = 48 hours when compared with t = 0. (N = 6 experiments were performed). **B.** Average total neurite length per RGC was evaluated by manually measuring RGC neurites in photo-micrographs acquired by live-cell imaging. (N = 6 experiments). Error bars, SD. Student’s *t* test. **p<0.01; *p<0.05.

We determined RGC survival on laminin and PDL by manually counting live RGC in photomicrographs acquired by live-cell imaging, and showed that RGCs exhibited 70% survival on laminin, in contrast to only 50% on PDL 48 hours after plating (Fig 5A).

Combined, these results suggest that RGCs adhesion to laminin induces integrin-mediated signaling with up-regulation of β1 integrin expression, and FAK activation, key regulators of the integrin survival signaling, and enhances RGC survival.

### β1 Integrin Activation Mimics the Laminin Survival Effect in RGCs

The monoclonal antibody HUTS-21 is a β1 integrin activating antibody that recognizes and binds to an activation-dependent epitope in the amino acid 355–425 region, and maintains β1 integrin in an activated state on the cell surface [16]. To examine the effect of β1 integrin activation, on integrin signaling and RGC survival, purified RGC were cultured on PDL, and treated 12 hours later with HUTS-21, or isotype control antibodies (1 µg/ml) for 24, or 48 hours. We performed immunohistochemistry with β1 integrin, FAK, and [pY397]-FAK antibodies and employed live cell imaging to determine RGC survival 48 hours after plating.

RGC cultured on PDL but treated with β1 integrin activating antibody, HUTS-21, exhibited a similar response to the exposure to laminin: increased β1 integrin expression (Fig 4 i and inset), and FAK activation by phosphorylation (Fig 4 k and inset). The increase in both markers was detected compared to RGC treated with an isotype control antibody as a reference (Fig 4 j and inset, respectively 4 l and inset). In addition β1 integrin agonist antibody HUTS-21 significantly enhanced RGC survival on PDL to the level observed for laminin, while an isotype control antibody didn’t have any effect on survival (Fig 5A). These results suggest that β1 integrin activation is sufficient to mimic laminin-mediated survival effect.

However, in contrast to laminin, the HUTS-21 antibody did not significantly affect RGC neurite outgrowth on PDL (Fig 5B).

### FAK is Downstream of β1 Integrin and Regulates RGC Survival and Neurite Growth

We next asked whether FAK is required for the survival effect of laminin, or HUTS-21 in RGCs. Pretreatment of cultured RGCs with PP2, a specific tyrosine kinase pharmacological inhibitor of Src/FAK, abolished the pro-survival effect of both laminin, and HUTS-21 antibody (Fig 5A). Thus, laminin and HUTS-21-mediated β1 integrin activation are sufficient to promote RGC survival through FAK-dependent signaling.

**Figure 6:**
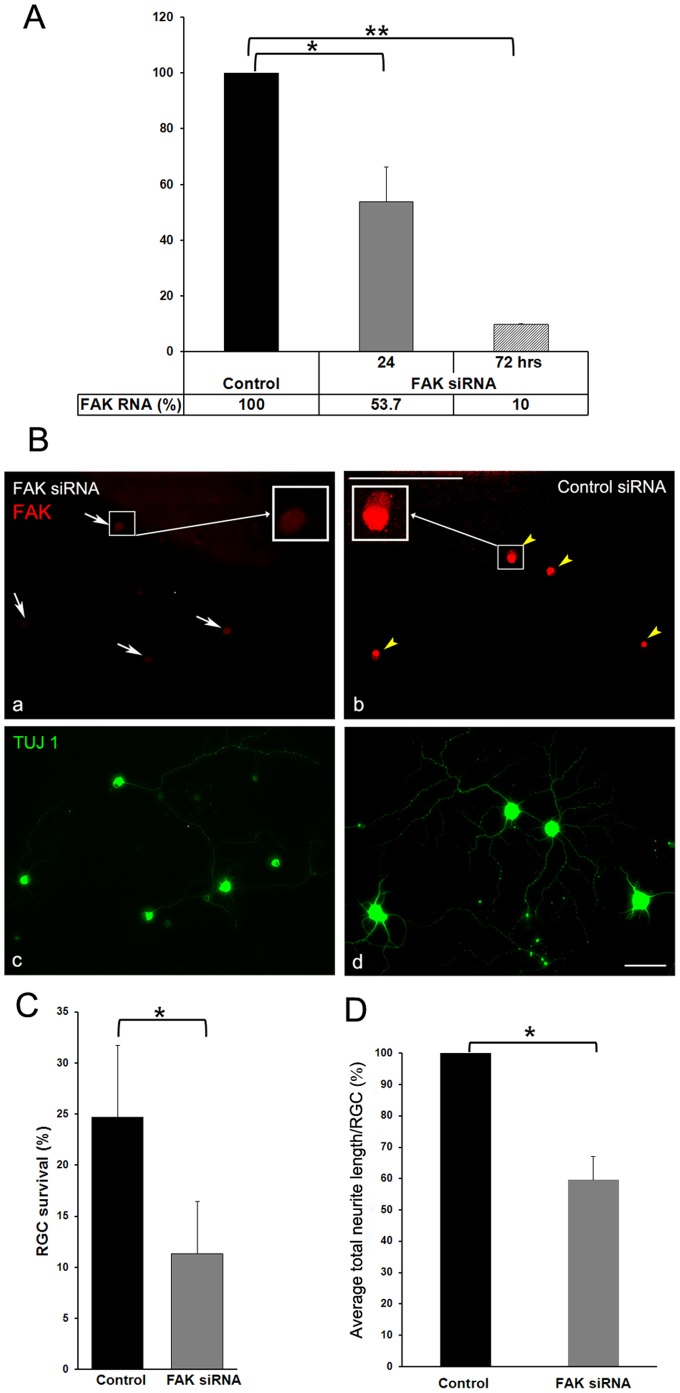
FAK regulates RGC survival, and neurite growth. P3 RGCs were transfected by electroporation with FAK siRNA and non-targeting control siRNA and plated on laminin for 72 hours. **A**. The knockdown efficiency of the FAK siRNA was verified by RT-PCR with specific FAK primers after 24, and 72 hours in culture. (N = 3 experiments). Error bars, SD. Student’s *t* test. **p<0.01; *p<.0.05. **B**. Double immunolabeling with FAK (red), and TUJ1 (neuronal class III β-tubulin) (green) antibodies in RGC 72 hours post-transfection showed almost complete depletion of FAK protein expression in RGC transfected with FAK siRNA (**a**, white arrows; **b**), whereas FAK expression is not affected in RGC transfected with non-targeting control siRNA (**c,** yellow arrowheads; **d**). Boxed insets in **a**, and **b** represent higher magnification images (63X) of a representative cell in each condition; n≥100 RGC per condition were analyzed. A representative experiment is shown. At least N = 3 independent experiments were performed. Scale bar: 10 µm. FAK siRNA knock-down resulted in (**C**) decreased RGC survival, and (**D**) reduced average total neurite length per RGC when compared with non-targeting siRNA control 72 hours post-transfection; n≥100 RGC per condition were analyzed for both survival and neurite length. N = 3 experiments were performed. Error bars, SD. Student’s *t* test. *p<0.05.

To further assess the role of FAK in RGC survival and neurite outgrowth, we used FAK siRNA to knock-down FAK expression in purified RGC. RT-PCR showed that FAK RNA expression was 90% reduced 72 hours after siRNA transfection (Fig 6A). Correspondingly, immunohistochemistry with anti-FAK antibodies showed greatly reduced expression of FAK in TUJ1 (neuronal class III β-tubulin) labeled RGC in culture (Fig 6B). FAK knock-down in RGC resulted in more than 2 fold decrease in RGC survival (Fig 6C), and 1.8 fold decrease in average total neurite length per RGC (Fig 6D). Collectively, these results indicate that FAK is a key down-stream effector of laminin-integrin signaling, and regulates RGC survival, and neurite outgrowth.

### HUTS-21 Enhances RGC Survival and Rescues Integrin Signaling *in vivo* after RIRI

We next explored whether the β1 integrin activating antibody, HUTS-21, could rescue RGC survival in vivo, in the rat RIRI model.

**Figure 7:**
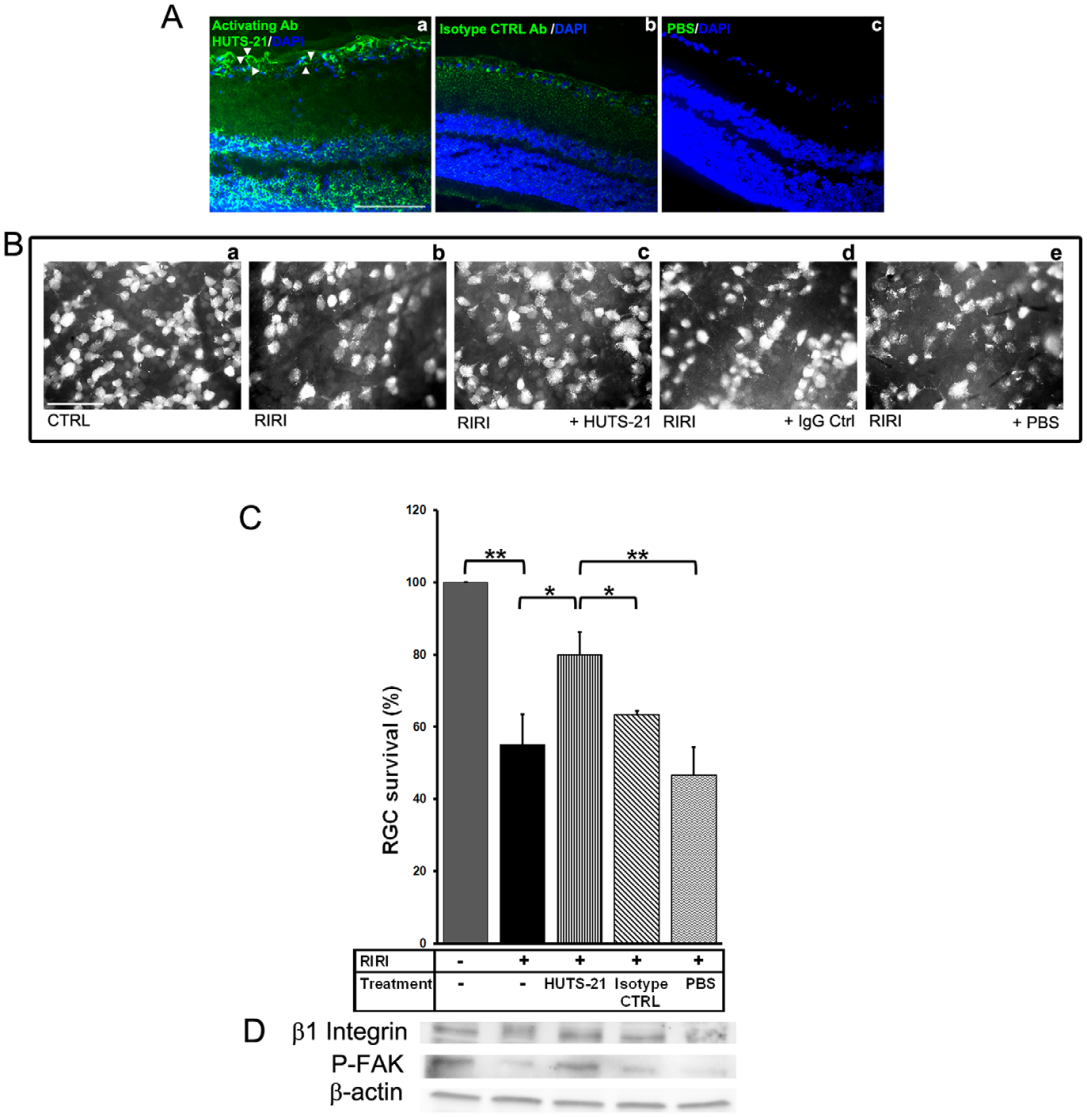
The β1 agonist antibody HUTS-21 enhances RGC survival and rescues β1 integrin-FAK signaling in vivo after RIRI. HUTS-21, or isotype control (CTRL) rat antibodies (0.5 mg/ml), or PBS were administered intravitreally (5 µl) in the rat eye twice, 30 minutes, and 2 days after RIRI. **A.** Immunostaining with a secondary anti-rat fluorescent antibody demonstrating localization of HUTS-21 antibody into the retina 24 hours after RIRI (n = 3 animals were analyzed). Note that HUTS-21 binds to activated β1 receptors on RGC cell body, dendrites, and axons (a, white arrowheads). **B.** Representative photo-micrographs of retinal flat-mounts of control rats (a), non-treated (RIRI) (b), and intravitreally treated with HUTS-21 (c), IgG control (IgG Ctrl) (d), and vehicle PBS (e) ischemic rats 5 days post-injury (n = 4–6 animals for each group). **C.** RGC survival was determined by counting FG retrogradely labeled RGCs in flat-mounted retinas 5 days post-RIRI (n = 4–6 animals for each group). Error bars, SD. Student’s *t* test. **p<0.01; *p<0.05. **D.** Western blot analysis of retinal extracts with β1 integrin, and P-FAK antibodies, 1 day post-RIRI (n = 4). A representative experiment is shown. N = 3 experiments.

Before testing the neuroprotective properties of HUTS-21, we asked if this antibody localizes to the retina after intravitreal injection. 5 µl of rat HUTS-21, or isotype control antibody (0.5 mg/ml), or vehicle control PBS were administered intravitreally to the rat eye. One day after injection, rats were euthanized, their retinas were cryo-sectioned, and immunohistochemistry with a secondary anti-rat fluorescent antibody was performed. We found strong fluorescent immunostaining in the retina of animals treated with HUTS-21, in the GCL, but also in INL, ONL, and retinal pigment epithelium (RPE) at 1 day post-injection. In GCL, HUTS-21 binds to activated integrin β1 receptors present on the RGC body, dendrites, and axons in the nerve fiber layer (NFL) (Fig 7A a). In correlation with previous studies [38]–[40], our immunohistochemistry with β1 integrin antibodies in retinal sections (Fig 3 b, c) showed that integrin β1 is widely expressed in the retina, in GCL, photoreceptors, RPE, and retinal blood vessels. In contrast, there was diffuse distribution of IgG control antibodies (Fig 7A b) throughout the retina, indicating that the antibodies cross ILM and reach the retina, but don’t bind specifically to β1 integrin receptors. There was no fluorescent staining in the retina after vehicle control PBS injection (Fig 7A c).

To test the neuroprotective properties of agonist antibody HUTS-21 we subjected rats to RIRI, injected intravitreally HUTS-21, or isotype control antibodies, or PBS vehicle twice, 30 minutes, and 2 days post-RIRI, and determined RGC survival 5 days post-injury. RIRI resulted in 45% RGC death (Figure 7B b, C), but HUTS-21 administration in vivo significantly reduced RGC death to only 20% (Fig 7B c, C). The isotype control antibody (Fig 7B d, C) or PBS control (Fig 7B e, C) had no significant effect on RGC survival. In addition, administration of HUTS-21 prevented the disruption of β1 integrin signaling in RGC, as evidenced by persistent β1 integrin expression and FAK phosphorylation 1 day post-injury (Fig 7D). Thus, stimulating β1 integrin activation in the context of laminin degradation normally observed after ischemic injury, partially restored integrin survival signaling, and prevented RGC loss in vivo.

## Discussion

### ECM Laminin is a Major RGC Survival Factor

The ECM protein laminin has been well-described in promoting RGC survival and neurite growth in vitro, and vivo [6], [24], [41]–[43]. We and others have shown that laminin degradation after retinal [7], or brain ischemia [44] is associated with decreased neuronal survival in those models. MMP-9 knockout mice or mice treated with MMP-inhibitors demonstrate reduced laminin degradation, and reduced neuronal death after injury [7], [44]–[47]. Oxidative stress results in disruption of focal adhesions (FA) in the retinal ganglion cell line RGC-5, and laminin protective effect against oxidative injury in vitro is mediated specifically by β1 integrin receptors (unpublished data).

In the present study, we explored the laminin downstream signaling and found that integrin survival pathway is disrupted in RGCs after RIRI. Prominent features include reduced β1 integrin expression, FAK, and Akt dephosphorylation, and a decrease in anti-apoptotic protein Bcl-xL expression. We characterized MMP-9 activation, laminin degradation, and RGC death in the rat RIRI model, and correlated these changes in a temporal, and spatial manner with changes in the β1 integrin survival pathway in RGCs. MMP-9 up-regulation was detected as early as 6 hours in GCL, clearly co-localized with RGC, but other sources of MMP-9, such as astrocytes [7], and infiltrating microglia, and neutrophils are present in ischemia [48]. MMP-9 increased expression and activity at 1 day post-ischemia correlated with laminin degradation, changes in the integrin signaling pathway in RGCs, and RGC apoptosis. These molecular changes preceded RGC death, which was minimal at 1 day, but reached 40% at 5 days post-injury.

It is interesting that laminin degradation occurs not only along the ILM, which borders RGC axons and cell bodies, but also in the INL, and inner plexiform layer (IPL), where amacrine and bipolar cells establish synapses with each other, and with RGCs. An important function of laminin surrounding the neuronal cell body and dendrites in regeneration has previously been suggested [49], but how such an effect translates to survival signaling is not known. The loss of laminin in the INL could contribute to a loss of electrical activity in RGCs [1]. We previously demonstrated that physiologic levels of electrical activity are potent stimuli for trophic responsiveness, survival, and axon growth in RGCs in vitro [6]; electrical stimuli may also prolong RGC survival after optic nerve injury in vivo [50]. Future studies exploring the loss of laminin in these inner layers and its effects on synaptic function, and retinal activity may be warranted, for example by specifically inhibiting laminin degradation in only one retinal layer after injury.

### Laminin-β1 Integrin-FAK Signaling Regulates RGC Survival

Laminin has been described to signal through a number of different receptors such as integrins and dystroglycan, to regulate survival, differentiation, cell shape and migration [51]–[53]. Our data suggest that β1 integrin is a critical mediator of RGC survival signaling, as β1 integrin expression is reduced in the period after ischemia, stimulating β1 integrins with activating antibodies enhanced RGC survival in vitro, and β1 integrin blocking antibodies inhibited laminin-induced survival signaling, and neurite outgrowth [41], [54]. This suggests that laminin survival signals are specifically mediated by β1 integrin receptors in RGCs.

Integrins are non-kinase receptors, therefore their binding to ECM, and subsequent activation requires kinases to initiate signal transduction. β1 integrin signaling occurs primarily through the recruitment and activation of the tyrosine protein kinase FAK [33], [34], [37], [55]. Alternatively, it may involve the threonine/serine integrin-linked kinase (ILK) as demonstrated in hippocampal neurons and brain endothelial cells [56]–[58], or the Src family kinase Fyn in oligodendrocytes [59]. FAK has previously been implicated in axonal outgrowth and guidance [60], [61] and synaptic plasticity [62], [63]. The non-receptor protein kinases Src, and FAK are two important cellular signaling components known to act cooperatively in transduction of survival signals [35], [37]. Our studies show that PP2, a specific Src/FAK inhibitor, inhibits the pro-survival effect mediated by both laminin, and β1 integrin activating antibody, suggesting that FAK is an important downstream regulator of β1 integrin. FAK siRNA knockdown resulted in reduced RGC survival and neurite outgrowth. FAK has an important role in prevention of apoptosis by cell attachment; FAK activation by phosphorylation is associated with survival, whereas FAK dephosphorylation is associated with apoptosis [64], [65]. After ischemia we observed a significant dephosphorylation of FAK in RGCs, while FAK expression didn’t change. Our results reveal an important role for FAK in regulating RGC survival and neurite outgrowth, and show that laminin survival signals are transmitted to integrins and integrated by FAK phosphorylation in RGCs.

PI3K/Akt and Ras/Raf/MEK/ERK are the most well-known cell-survival promoting pathways engaged in mediating FAK/Src signaling by integrins [14], [33]. Associated with decreased FAK phosphorylation after ischemic injury, we observed a decrease in Akt phosphorylation and anti-apoptotic protein Bcl-xL expression as early as 1 day post-RIRI. These findings are consistent with previous studies reporting that integrin-mediated cell survival implicates PI3K/Akt and results in up-regulation of anti-apoptotic bcl family members [36], [66], [67]. In addition, specific deletion of laminin γ1 in Schwann cells results in decreased cell survival [68] and in cortical neurons affects neurite outgrowth and migration [69] through disruption of a similar signaling pathway, via integrin-FAK-Akt/GSK-3β.

PI3K-dependent Akt phosphorylation and subsequent increased synthesis of anti-apoptotic bcl proteins represents a common mechanism of regulating RGC survival shared by many growth factors such as BDNF, IGF, CNTF, HGF [23], [70]–[73], and cytokines such as IL-1β and erythropoietin [74], [75]. Recent studies have shown that the Akt isoforms, 1, 2, and 3 are present in the RGC layer in the rat retina, and all three subtypes are able to block RGC apoptosis after RIRI [76]. In the future, it might be interesting to study the differential role of Akt subtypes in laminin-integrin mediated survival signaling after RIRI.

**Figure 8:**
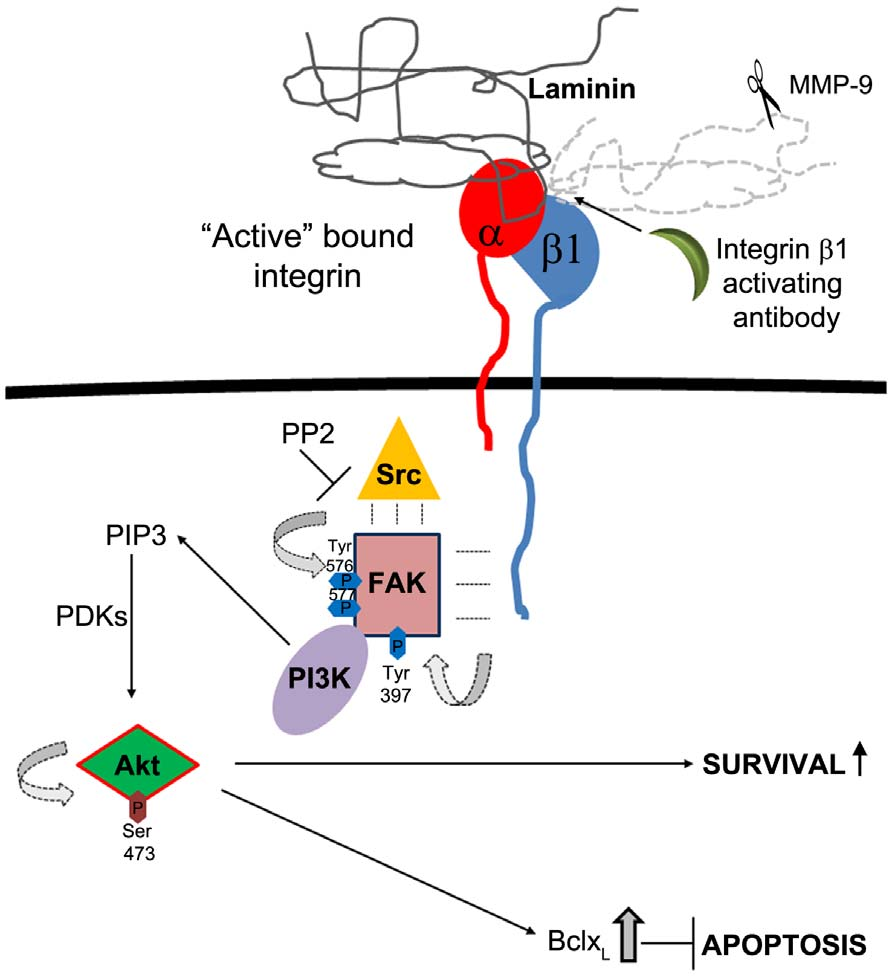
Schematic diagram of laminin-integrin survival signaling in RGC. RGC adhesion to laminin induces integrin activation and initiates integrin signaling. Integrin β1 undergoes a conformational change to an “active” high affinity bound state [83]. Alternatively, in the absence of laminin, or in the context of MMP-9 mediated laminin degradation observed after RIRI *in vivo*, a β1 integrin stimulatory antibody can bind and maintain integrin β1 in the “active”, signaling conformation at the RGC’s membrane. Activated β1 integrin initiates signal transduction by recruiting the tyrosine protein kinase, FAK. In response to integrin engagement, FAK auto-phosphorylates at Tyr 397 which represents the initial, major step in FAK activation. FAK P-Tyr 397 creates a binding site for Src family kinases. Src recruitment, and activation leads to phosphorylation of multiple amino acids including Tyr 576, 577 required for FAK’s full catalytic activity [37]. PP2 inhibits Src, therefore FAK’s complete activation required for downstream survival signaling. Initial Tyr 397 phosphorylation is also important for recruitment of PI3K. FAK promotes cell survival downstream of laminin and integrin β1 by enhancing PI3K mediated activation of Akt by phosphorylation at Ser 473. Activated Akt is associated with survival [35], [37], and inhibition of apoptosis by increasing expression of anti-apoptotic protein bclx_L_.

Taken together, our in vivo experiments in the rat RIRI model, and in vitro in RGC in culture, demonstrate that β1 integrins translate RGC adhesion to laminin into survival signals mediated by FAK, and associated with Akt activation, and increased expression of the anti-apoptotic protein Bcl-xL (Fig 8).

### β1 Integrin Stimulatory Antibody HUTS-21 Reduces RGC Death and Rescues β1 Integrin Survival Signaling after Ischemia-reperfusion Injury

Functional efficacy of integrins depends not only on their elevated expression at the cell surface [77]–[79], but also on their level of activation [80]–[82]. Integrin activation involves a conformational change from an “inactive”, low ligand binding affinity state, to an “active” state, with high ligand binding affinity [83]. Low ligand binding affinity, inactive integrins might be responsible for low regenerative ability of neurons in adult CNS, where the levels of growth promoting laminin are low [81], [84]. Several strategies have been proposed to modulate integrin activation; integrins can be activated “outside-in” by ECM ligand binding, increase in extracellular ion concentrations, such as manganese (Mn^2+^), and monoclonal antibodies [81], [85]. For instance, the monoclonal antibody TASC promotes retinal cells’ adhesion to laminin [86], while the activating monoclonal antibody TS2/16 alleviates the inhibitory effect of aggrecan on axon outgrowth in dorsal root ganglia neurons [85]. On the other hand, treatment with phorbol esters [87], introduction of a constitutively activated Ras, R-ras^38V^, or pharmacological increase of c-AMP results in “inside-out” integrin β1 activation, in correlation with increased laminin adhesion, and enhanced neurite outgrowth of retinal neurons [81], [82]. The β1 monoclonal activating antibody, HUTS-21, recognizes selectively only the active conformation of β1 integrin; once it recognizes, binds to it and maintains integrin activated at the surface of the cell [16]. HUTS-21 was used successfully as a therapeutic agent in a model of rat renal ischemia reperfusion injury to maintain β1 activated at the surface of tubular cells, to preserve renal function, and to prevent tissue damage after ischemic injury [88]. Our data shows that HUTS-21 promoted RGC survival in vitro to a level similar to laminin. Interestingly, in contrast to laminin, HUTS-21 does not promote neurite extension, indicating that different regulatory sequences in the integrin molecule, or conformations might be responsible for survival, and neurite extension. Here, we report the neuroprotective effect of intravitreal administration of HUTS-21 in a rat RIRI model. These data suggest that maintaining β1 integrin in an activated state may prevent RGC death after ischemia-reperfusion injury as occurs in retinal artery or vein occlusions, and HUTS-21 antibody could also be explored as a neuroprotective agent for the treatment of ischemic optic neuropathy and glaucoma. Because transient-global-cerebral-and retinal ischemia share similar mechanisms [17], [89], our studies also have relevance to stroke.
